# Vagus Nerve Stimulation Attenuates Cerebral Microinfarct and Colitis-induced Cerebral Microinfarct Aggravation in Mice

**DOI:** 10.3389/fneur.2018.00798

**Published:** 2018-09-26

**Authors:** Xiaofeng Chen, Xiaofei He, Shijian Luo, Yukun Feng, Fengyin Liang, Taotao Shi, Ruxun Huang, Zhong Pei, Zhendong Li

**Affiliations:** ^1^Department of Neurology, The Fifth Affiliated Hospital, Sun Yat-sen University, Zhuhai, China; ^2^Department of Neurology, National Key Clinical Department and Key Discipline of Neurology, The First Affiliated Hospital, Sun Yat-sen University, Guangzhou, China

**Keywords:** cerebral microinfarct, colitis, vagus nerve stimulation, blood-brain barrier, inflammation, oxidative stress

## Abstract

Cerebral cortical microinfarct (CMI) is common in patients with dementia and cognitive decline. Emerging studies reported that intestinal dysfunction influenced the outcome of ischemic stroke and that vagus nerve stimulation (VNS) protected against ischemic stroke. However, the effects of intestinal dysfunction and VNS on CMI are not clear. Therefore, we examined the influence of colitis and VNS on CMI and the mechanisms of VNS attenuating CMI in mice with colitis. CMI was induced using a two-photon laser. Colitis was induced using oral dextran sodium sulfate (DSS). The cervical vagus nerve was stimulated using a constant current. *In vivo* blood-brain barrier (BBB) permeability was evaluated using two-photon imaging. Infarct volume, microglial and astrocyte activation, oxidative stress and proinflammatory cytokine levels were assessed using immunofluorescent and immunohistochemical staining. The BBB permeability, infarct volume, activation of microglia and astrocytes and oxidative stress increased significantly in mice with colitis and CMI compared to those in mice with CMI. However, these processes were reduced in CMI mice when VNS was performed. Brain lesions in mice with colitis and CMI were significantly ameliorated when VNS was performed during the acute phase of colitis. Our study demonstrated that VNS alleviated CMI and this neuroprotection was associated with the suppression of BBB permeability, neuroinflammation and oxidative stress. Also, our results indicated that VNS reduced colitis-induced microstroke aggravation.

## Introduction

Cerebral cortical microinfarct (CMI) has gained increasing attention because of its major role in cognitive impairment and dementia (1). Recent autopsy studies reported that CMI were detected not only in patients with dementia (62% in vascular dementia and 43% in Alzheimer's disease), but also in older individuals without dementia (up to 33%) (1, 2). However, effective therapies for CMI are lacking.

Inflammatory bowel disease (IBD), which includes Crohn's disease and ulcerative colitis, is a group of chronic relapsing intestinal inflammatory disorders. Recent evidence demonstrated that intestinal dysfunction aggravated poststroke neuroinfla- mmation and outcome in experimental stroke (3, 4). A cohort study also demonstrated a greater than two-fold increased risk of atrial fibrillation (AF) during IBD flares and persistent activity (5), and AF is an important risk factor of CMI (6). Many studies reported that IBD was a contributor to an increased risk of ischemic heart disease and cerebrovascular accidents during periods of active disease (7, 8). Therefore, we hypothesized that IBD would impact CMI, which, to our knowledge, has not been examined.

The vagus nerve is a link between the brain and gut. Previous studies demonstrated that vagus nerve stimulation (VNS) reduced infarct volume and improved neurological score and motor function recovery after ischemic brain injury (9–12), but the mechanism of these effects remains controversial. Recent evidence demonstrated that VNS conferred neuroprotection against ischemic brain injury via the cholinergic anti-inflammatory pathway (CAIP) (13). Other studies reported that VNS reduced the blood-brain barrier (BBB) permeability and cerebral ischemia/reperfusion (I/R) injury (14, 15). However, the effect of VNS on CMI is not known. The anti-inflammatory effects of VNS against intestinal inflammation are well documented (16–19).

The present study examined the effect of VNS on CMI in mice with or without colitis induced via dextran sodium sulfate (DSS) administration. We also investigated the mechanisms of protective role of VNS.

## Materials and methods

### Animals

**Figure 1:**
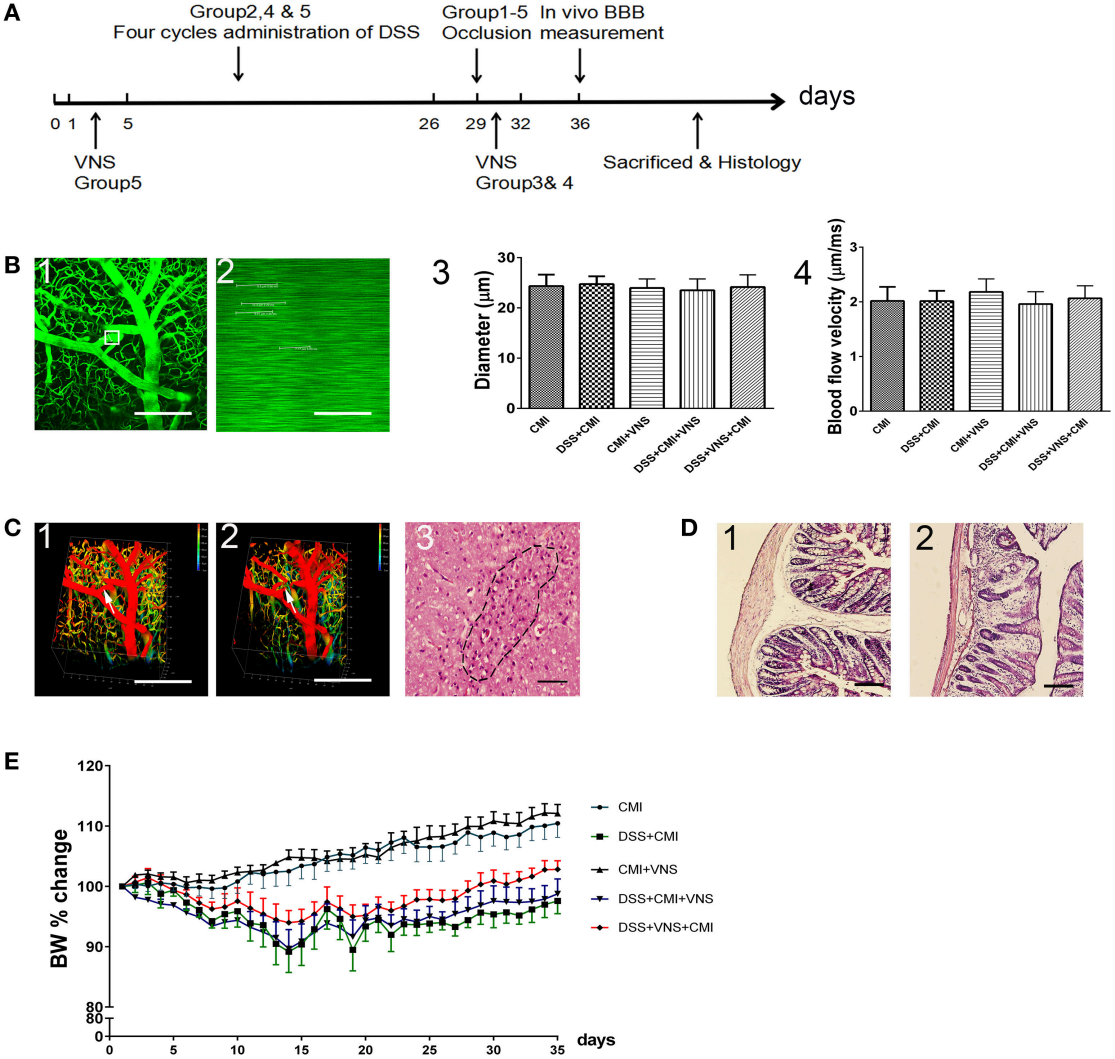
Establishment of CMI and DSS-induced colitis models. **(A)** Timeline for different groups (*n* = 8 per group). Group 1: CMI mice. Group 2: DSS+CMI mice. Group 3: CMI+VNS mice. Group 4: DSS+CMI+VNS mice. Group 5: DSS+VNS+CMI group. **(B1)** Candidate arteriole for clotting was identified, and microvessel diameter was measured. **(B2)** Red blood cell speed was measured prior to clot formation. **(B3,B4)** Microvessel diameter and red blood cell speed in each groups. **(C1, C2)** *in vivo* three-dimensional stacks of images of the target vessels through the cranial window before and after occlusion (white arrow). **(C3)** H&E staining of brain section in CMI model (200×). **(D1, D2)** Immunohistochemical analyses of intestinal inflammation (H&E, 100×). **(E)** Cumulative changes in body weight (BW) in each group. Scale bars, 200 μm.

The Sun Yat-sen University (Guangzhou, China) Committee on the Care and Use of animals approved all animal experiments. Experiments were performed in a blinded manner. Forty wild-type C57BL/6J female mice (8–10 weeks, 20–25 g) were used. We employed females only according to previous study, because males are more sensitive to the disruptive effects of DSS on colon epithelia and can develop severe inflammation and die (20). All mice were purchased from the Animal Center of Sun Yat-sen University and housed at controlled temperature and humidity under a 12-h light/dark cycles. Free access to food and water was supplied. All animals were randomly assigned to five groups. The CMI group mice underwent occlusion of a single penetrating arteriole only. The DSS+CMI group mice received four cycles of DSS followed by occlusion of a single penetrating arteriole on day 29. The CMI+VNS group mice received VNS 30 min, 1, 2, and 3 days after CMI induction. The DSS+CMI+VNS group mice underwent four cycles of DSS followed by occlusion of a single penetrating arteriole on day 29, and VNS was performed 30 min, 1, 2, and 3 days after the induction of CMI. The DSS+VNS+CMI group mice underwent four cycles of DSS, and VNS was performed during the acute phase of colitis followed by occlusion of a single penetrating arteriole on day 29. Figure 1A presents the study design.

### Experimental colitis

Experimental colitis was induced using multiple-cycle administration of 2% (wt/vol) DSS (molecular weight 30,000 to 50,000, MP Biomedicals, CANADA) in drinking water on days 1 to 5, 8 to 12, 15 to 19, and 22 to 26, as previously described (20, 21). Drinking water with fresh DSS solutions was replaced daily. Control mice received drinking water without DSS. Bodyweights were monitored daily.

### Electric VNS

The stimulating electrodes were self-constructed based on the design of Smith et al. (22). The electrodes were composed of two polyethylene-coated curved silver wires held 1.5 mm apart with a solid bar. Briefly, the mice were anesthetized via an intraperitoneal injection of pentobarbital (1%, 50 mg/kg), and an incision was performed in the ventral side of the neck to isolate the left cervical vagus nerve. Bipolar electrodes were gently wrapped around the vagus nerve using a microscope and sutured to the sternocleidomastoid muscle. The mice in the treatment group were subjected to VNS (0.5 mA, 5 Hz) using a stimulator (Model SDZ-II, Medical Appliance Factory, Suzhou, China). Stimulation was delivered for 30 s every 5 min for 1 h. The mice in the sham group underwent the same procedure but did not receive stimulation. Each mouse was given gentamicin (4 mg/kg) to prevent infection only immediately after completion of surgery. For CMI mice and DSS+CMI+VNS mice, VNS was performed 30 min, 1, 2 and 3 days after the onset of CMI. For DSS+VNS+CMI mice, VNS was performed during the acute phase of colitis (days 1 to 5) (10, 15, 16).

### Surgical procedures of the CMI model

The creation of micro-lesions was done in a blinded manner, without knowledge of animal assignment to groups. The mice were anesthetized via intraperitoneal injection of pentobarbital (1%, 50 mg/kg) and positioned in a stereotaxic frame (RWD Life Science Company, Shenzhen, China). A 2 × 2 mm^2^ cranial window was created using a microdrill over the right parietal cortex (2 mm bregma, 1.7 mm lateral), and a metal plate was glued to the edge of the cranial window for *in vivo* two-photon imaging, as described previously (23). Artificial cerebrospinal fluid (ACSF) was used to keep the window moist during the entire surgical procedure. The experiment was not performed if any bleeding was present in the cranial window. Body temperature was maintained at 36.8°C throughout the surgical procedure using a feedback-regulated heat pad (RWD Life Science Company, Shenzhen, China). Fluorescein isothiocyanate (FITC)-dextran (0.2 ml, 2,000 kDa, 2%; FD20, Sigma, Germany) was injected into the tail vein prior to mouse fixation on the stage of the two-photon laser scanning microscope (Leica, Germany). A 0.12 numerical aperture × 4 air objective was used to obtain three-dimensional stack images of the cerebral vessels through the cranial window. A 0.95 numerical aperture and × 25 water immersion objective was used for high-resolution imaging, measurements of vessel diameter and blood flow speed, and vessel occlusion. A penetrating arteriole, approximately 20–25 μm in diameter, was selected as the target vessel. The target arteriole was placed in the center of the screen prior to occlusion. A segment that coursed parallel to the cortical surface was magnified to allow the easy verification of clot formation. The bleach mode was used to damage the endothelium. These bleach points were located within the vessel lumen a few micrometers from the vessel wall. Irradiation was initiated using an 800 nm laser. The intensity (max. power 3.5 W) was controlled at 30% (1.05 W) in the EOM setting and was depth-dependent. The energy was gradually increased by 10% until extravasation of fluorescently labeled plasma was observed outside of the vessel lumen (24, 25). Irradiation was continued until a clot was visualized and the motion of red blood cells ceased. We observed this area for approximately 1 h until the downstream flow was redistributed and stabilized. Irradiation was repeated until the target vessel stopped flowing if the occlusion recanalized. Models with hemorrhage or diffuse burning were discarded. The diameters of cortical CMI in post-mortem studies of patients with dementia are 0.1–1 mm (25). The lesions in the present study were typically microscopic (<1 mm).

### Analysis of the BBB permeability

The BBB permeability was analyzed 1 week after occlusion of a penetrating arteriole. Rhodamine B (RD; 0.2 ml of 1% in saline; Sigma) was administered intravenously immediately prior to two-photon imaging to visualize the vasculature. Fluorescent intravascular dyes were used to measure leakage from the vasculature (26, 27). The total fluorescence intensity in the extravascular compartment was analyzed (28). Images (512 × 512 pixels) were acquired using a 0.95 numerical aperture and × 25 water immersion objective 0, 5, 15, 30, and 60 min after administration of fluorescent dextran, and XYZ stacks of images were obtained.

### Histology

Mice were perfused transcardially with saline followed by a 4% paraformaldehyde / 0.1 M PB solution (pH 7.4) after the BBB measurements. The right parietal cortex and colon were rapidly removed. The cortex was sandwiched between two glass slides separated by a distance of 2.5 mm to ensure a flat section. Sections (1 cm) of the distal colon were cut, cleared of feces, and washed with a 0.1 M PB solution. Tissues were postfixed overnight in the same fixative and cryoprotected in 20 and 30% sucrose/0.1 M PB solutions at 4°C. Brain and colon tissues were serially sectioned (10-μm thick) using a frozen microtome (Leica, Germany). Colon sections were stained with hematoxylin and eosin (H&E) for pathomorphological examination. Brain sections were treated for immunofluorescent and immunohistochemical staining. Slices for immunofluorescent staining were blocked in 0.3% Triton X-100 and 10% goat serum for 1 h at room temperature. Slices were incubated overnight at 4°C with the following primary antibodies: monoclonal mouse anti-mouse NeuN IgG (1:200, Millipore, USA), polyclonal rabbit anti-mouse NeuN IgG (1:500, Abcam, UK), polyclonal rabbit anti-mouse Iba1 IgG (1:500, Wako, Japan), polyclonal rabbit anti-mouse GFAP IgG (1:500, Abcam, UK) and mouse anti-mouse mouse 3-nitrotyrosine (NT) IgG (1:100, Abcam, UK). Slices were subsequently incubated with species-specific secondary antibodies for 1 h at room temperature. All slides were stained with DAPI and sealed using a glass coverslip. Sections for immunohistochemical staining were treated with 3% hydrogen peroxide for 20 min and 0.3% Triton and 10% goat serum for 1 h at room temperature. The sections were incubated with primary antibodies (1:300 anti-TNF-α, Wako, Japan) overnight at 4°C, followed by secondary antibodies for 1 h and then by diaminobenzidine in PBS (1:100) for several seconds. The sections were incubated with hematoxylin for nuclear staining. The sections were immersed in ethyl alcohol and dimethylbenzene for several minutes, and the slices were mounted using Permount TM Mounting Medium under a cover glass. Image analysis was performed using ImageJ software.

### Statistical analysis

All data are presented as the means ± standard deviations of the means (SD). The 3D image overlays were visualized using Leica Application Suite (LAS) Advanced Fluorescence Lite software (LAS AF Lite, 2.4.1 build 6384, Leica). ImageJ software (National Institutes of Health, Bethesda, MD, USA) was used to analyze the immunohistochemistry and immunofluorescent results. An experimenter who was blinded to the experimental condition analyzed all sections. A two-way repeated measures ANOVA with multiple comparisons was performed to compare the BBB permeability measurements. An independent-samples *t* test was used for dual comparisons. Means were compared using one-way ANOVA analysis followed by a *post hoc* Tukey's multiple comparison test for multiple comparisons. Statistical analyses were completed using GraphPad Prism software (GraphPad Software, La Jolla, CA, USA). A *P* value < 0.05 was considered statistically significant.

## Results

### Occlusion of a single penetrating arteriole and DSS-induced colitis

We used two-photon microscopy to select target penetrating arterioles in mouse cortex and measured the microvessel diameter (Figure 1B1) and red blood cell speed (Figure 1B2) prior to clot formation. The average microvessel diameter (Figure 1B3) and red blood cell speed (Figure 1B4) did not differ statistically between each group (all *Ps* > 0.05). We used femtosecond laser ablation to trigger clotting in the selected penetrating arterioles (Figures 1C1,C2). The brain tissue injury caused by the lesion was visualized by H&E staining (Figure 1C3). Histological changes in DSS-induced intestinal inflammation were analyzed using H&E staining of colonic sections (Figures 1D1,D2). All mice exhibited signs of intestinal inflammation as evidenced by infiltration of inflammatory cells, loss of crypt structure and depletion of goblet cells (Figure 1D2). Body weight was monitored daily. A loss of 8 to 15% body weight was observed initially in DSS-treated mice, and normal weight was restored by day 28. Comparison between DSS+CMI group and DSS+VNS+CMI group indicated that VNS reduced the degree of body weight loss (Figure 1E).

### The effect of VNS on BBB disruption

A two-way repeated measures ANOVA indicated a significant interaction between the group and time factor (*P* < 0.001), and significant main effects of the time factor (*P* < 0.001) and group factor (*P* < 0.001) in all groups. The average fluorescence intensity in the extravascular compartment increased in the DSS+CMI group compared to that in the CMI group (*P* < 0.001) and decreased in the CMI+VNS group (*P* < 0.001) 60 min after RD injection, which indicates that the colitis worsened the BBB disruption and that VNS improved the BBB integrity after CMI.

**Figure 2:**
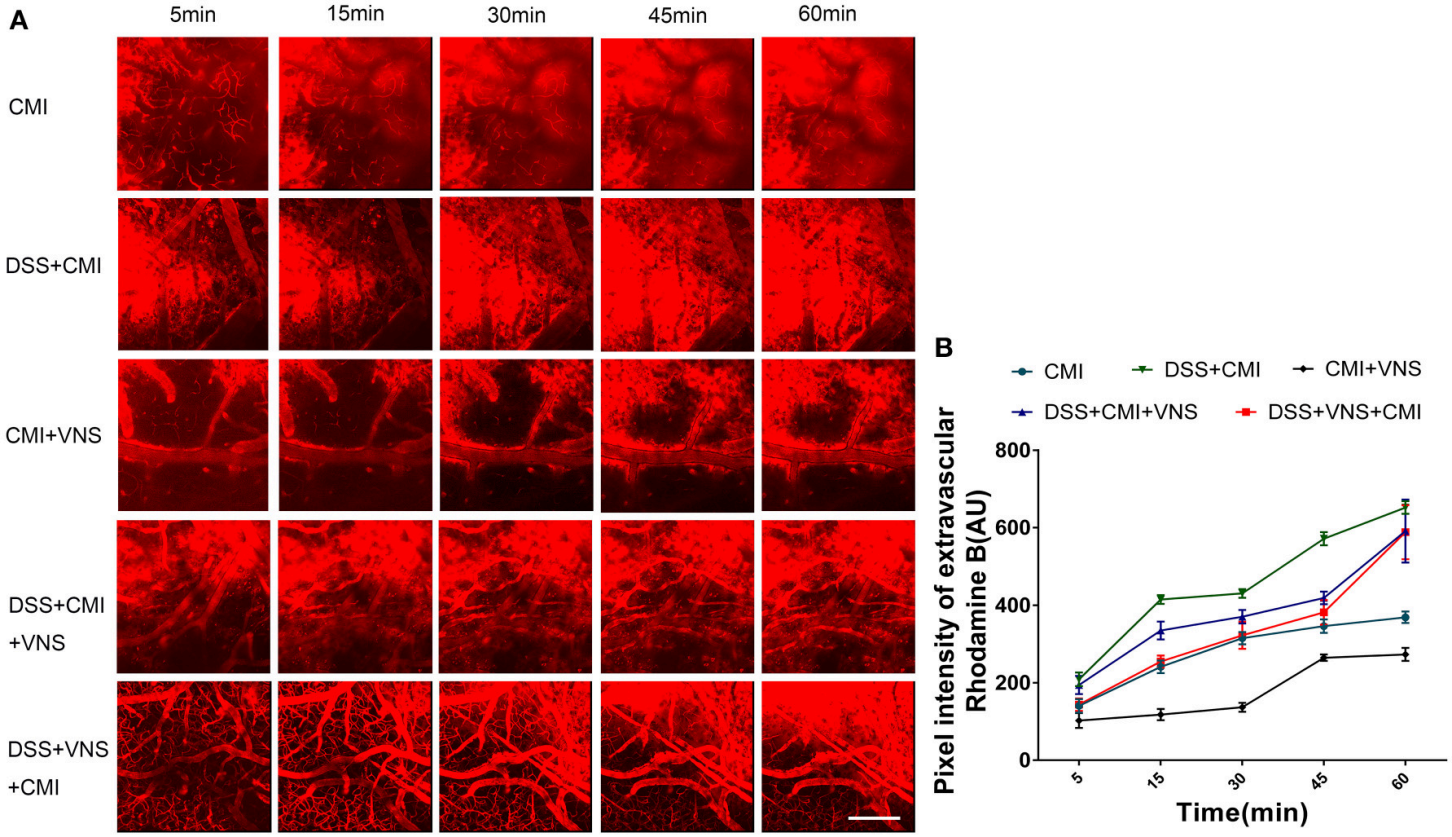
Analyses of two-photon microscopy data capturing fluorescent dye leakage of the BBB. **(A)** XYZ stacks of brain vessels 5, 15, 30, 45, and 60 min after dye injection (250×), indicating dye permeation. **(B)** Linear graph of comparison of average fluorescent intensities in the extravascular compartment at different time points (*n* = 8 per group). Scale bars, 200 μm.

The BBB disruption was attenuated in the DSS+VNS+CMI (*P* < 0.01) and DSS+CMI+VNS groups (*P* < 0.01) at 60 min compared with that of the DSS+CMI group, which indicates that VNS alleviated the BBB disruption in mice with CMI and colitis. We compared the BBB permeability of the DSS+CMI+VNS group and DSS+VNS+CMI group to assess the timing of VNS intervention and found that the magnitude of the decrease in the BBB permeability was more significant in the DSS+VNS+CMI group than in the DSS+CMI+VNS group at 5 min (*P* < 0.05), 15 min (*P* < 0.001) and 30 min (*P* < 0.05). However, there was no significant difference between the two groups at 45 min or 60 min (*P* > 0.05) (Figures 2A,B).

### The effect of VNS on infarct volume

**Figure 3:**
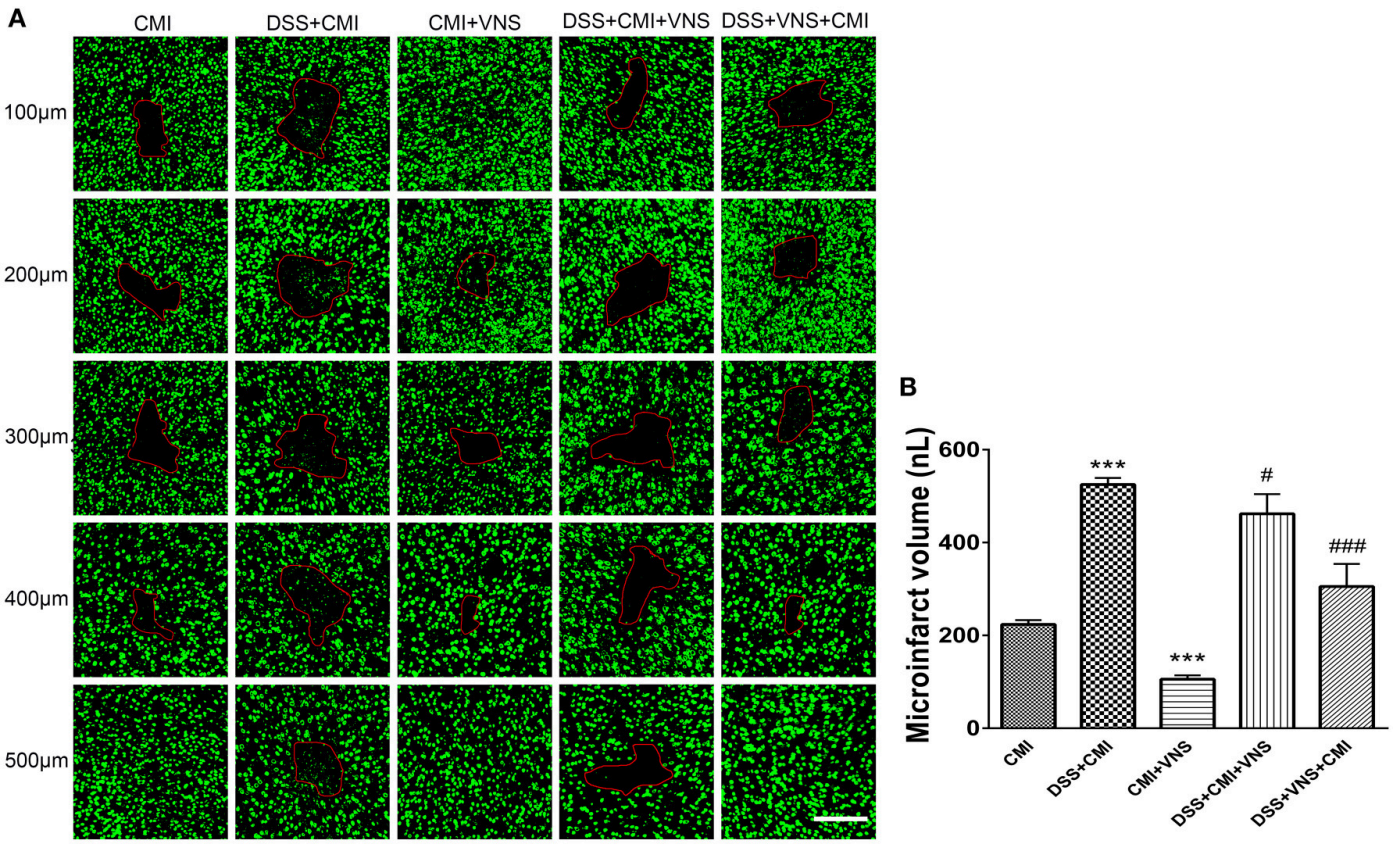
Measurement of CMI volume. **(A)** Total infarct volumes in the CMI, DSS+CMI, CMI+VNS, DSS+CMI+VNS, and DSS+VNS+CMI mouse groups. (200×) **(B)** Histograms comparing the infarct volume in the cortex (*n* = 8 per group). ^***^P ≤ 0.001 vs. CMI group. #*P* < 0.05 and ###*P* ≤ 0.001 vs. DSS+CMI group. Scale bars, 200 μm.

Experimental colitis significantly enlarged the infarct volume in the DSS+CMI group compared to that of the CMI group (223.658 ± 9.430 μm^3^ vs. 524.843 ± 14.262 μm^3^, *P* < 0.001). VNS treatment significantly reduced infarct volume in CMI mice (223.658 ± 9.430 μm^3^ vs. 105.567 ± 8.275 μm^3^, *P* < 0.001). We compared the DSS+CMI, DSS+VNS+CMI and DSS+CMI+VNS groups to evaluate the effect of VNS on CMI mice with colitis. We found a significant decrease in infarct volume in the DSS+VNS+CMI group (305.425 ± 48.406 μm^3^ vs. 524.843 ± 14.262 μm^3^, *P* < 0.001) and DSS+CMI+VNS group (461.825 ± 42.469 μm^3^ vs. 524.843 ± 14.262 μm^3^, *P* < 0.05). VNS treatment produced a greater improvement in the infarct volume in the acute phase of colitis than after the CMI onset (305.425 ± 48.406 μm^3^ vs. 461.825 ± 42.469 μm^3^, *P* < 0.05) (Figures 3A,B).

### The effect of VNS on activation of microglia and astrocytes

**Figure 4:**
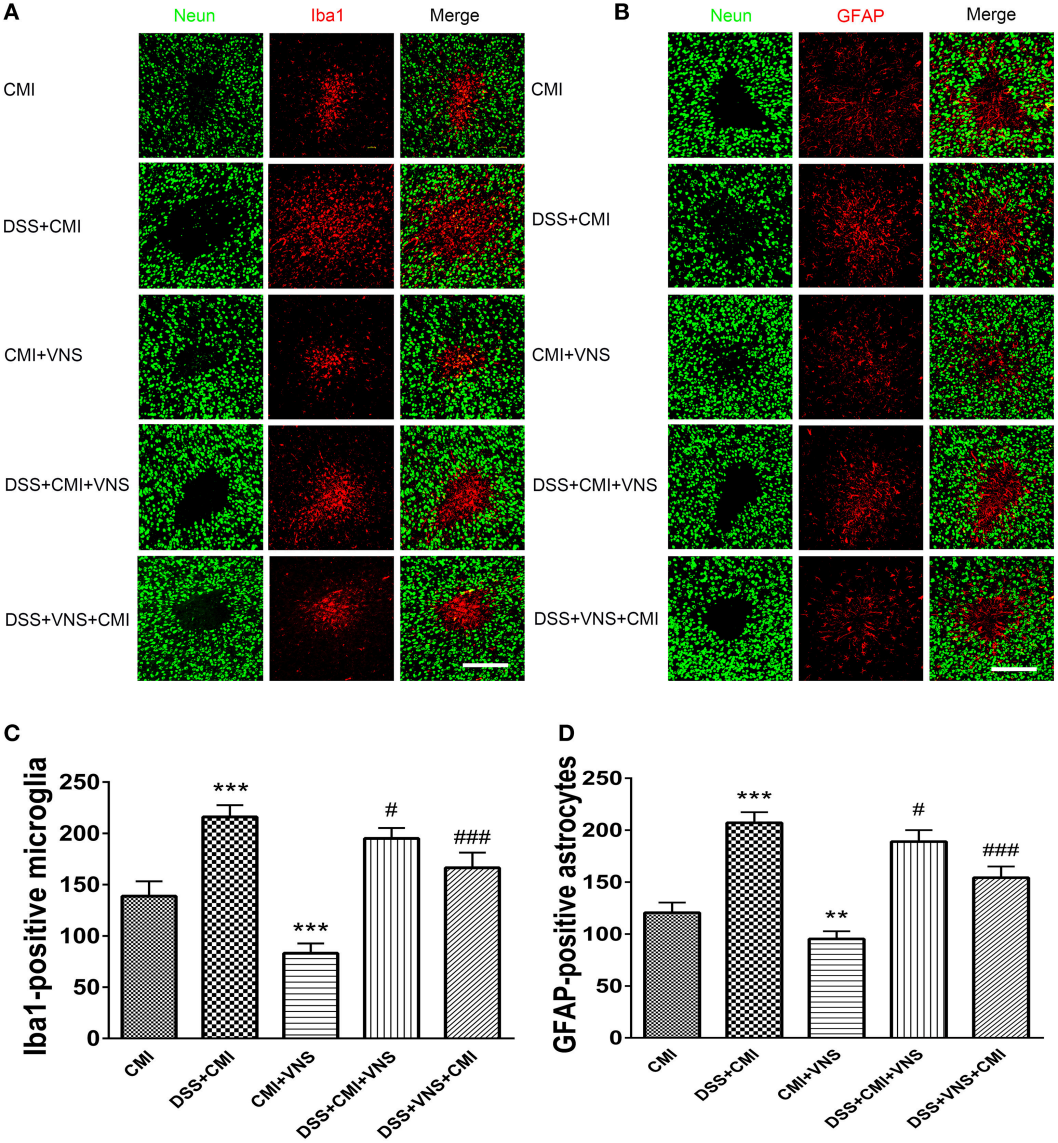
Measurement of activated microglia and astrocytes. **(A)** Immuno- fluorescent staining of ionized calcium-binding adapter molecule-1 (Iba-1)-positive microglia in the cortex of the CMI, DSS+CMI, CMI+VNS, DSS+CMI+VNS, and DSS+VNS+CMI mouse groups (200×). **(B)** Immunofluorescent staining of glial fibrillary acidic protein (GFAP)-positive astrocytes in the cortex of the CMI, DSS+CMI, CMI+VNS, DSS+CMI+VNS, and DSS+VNS+CMI mouse groups. (200×) **(C)** Histograms comparing the numbers of Iba1-positive microglia in the cortex (*n* = 8 per group). ^***^P ≤ 0.001 vs. CMI group. #*P* < 0.05 and ###*P* ≤ 0.001 vs. DSS+CMI group. **(D)** Histograms comparing the numbers of GFAP-positive astrocytes in the cortex (*n* = 8 per group). ^**^P < 0.01 and ^***^P ≤ 0.001 vs. CMI group. #*P* < 0.05 and ###*P* ≤ 0.001 vs. DSS+CMI group. Scale bars, 200 μm.

The DSS+CMI group exhibited greater numbers of Iba1+ cells in the infarct core (*P* < 0.001) and GFAP+ cells in the peri-infarct regions than those in the CMI group (*P* < 0.001), which indicates that colitis enhanced neuroinflammation in the acute phase of CMI. Our data suggest that VNS reduced Iba1+ cell infiltration and GFAP+ gliosis in CMI (*P* < 0.001 for Iba1+ cells and *P* < 0.01 for GFAP+ cells). Similar effects were observed in mice with colitis (DSS+CMI+VNS and DSS+VNS+CMI groups). Our results revealed that stimulation of the cervical vagus nerve was more effective during the acute phase of colitis than after the CMI onset (DSS+VNS+CMI group vs. DSS+CMI+VNS group, *P* < 0.05) (Figures 4A–D).

### The effect of VNS on deposition of NT

**Figure 5:**
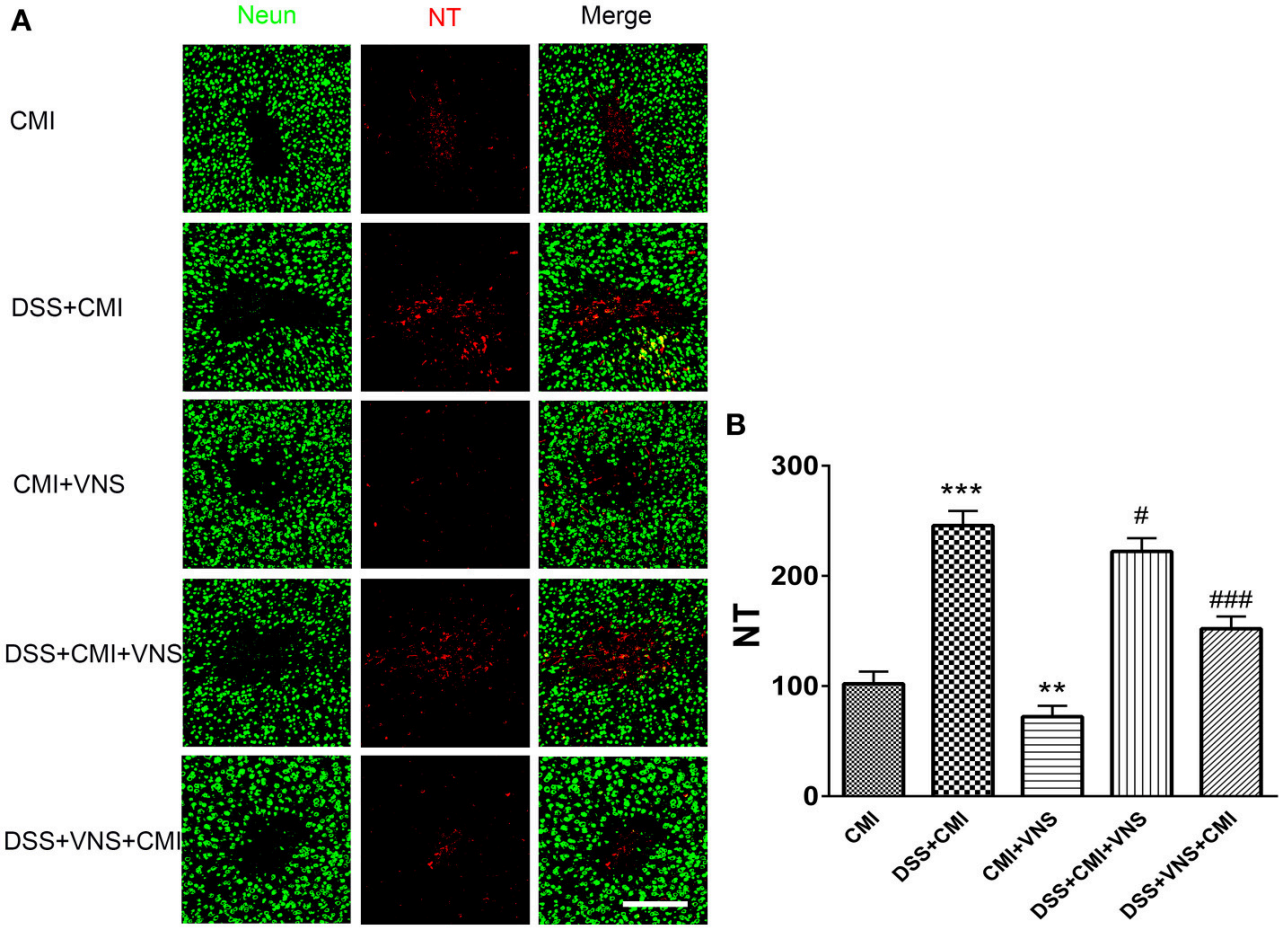
Measurement of NT deposition. **(A)** Immunofluorescent staining of NT deposition in the cortex of the CMI, DSS+CMI, CMI+VNS, DSS+CMI+VNS, and DSS+VNS+CMI mouse groups (200×). **(B)** Histograms comparing the numbers of 3-NT deposition in the cortex (*n* = 8 per group). ^**^P < 0.01 and ^***^P ≤ 0.001 vs. CMI group. #*P* < 0.05 and ###*P* ≤ 0.001 vs. DSS+CMI group. Scale bars, 200 μm.

DSS-induced colitis enhanced NT expression (*P* < 0.001), and VNS reduced NT expression compared to that of CMI mice (*P* < 0.01). VNS prior to CMI reduced NT deposition in DSS+CMI mice (*P* < 0.001), and VNS following the ischemic injury produced a mild effect on NT expression (*P* < 0.05). Comparison of the DSS+VNS+CMI and DSS+VNS+CMI groups revealed that NT expression decreased more in the former group, which suggests that VNS was more effective in the DSS+VNS+CMI group (*P* < 0.05) (Figures 5A,B).

### The effect of VNS on proinflammatory cytokine levels

**Figure 6:**
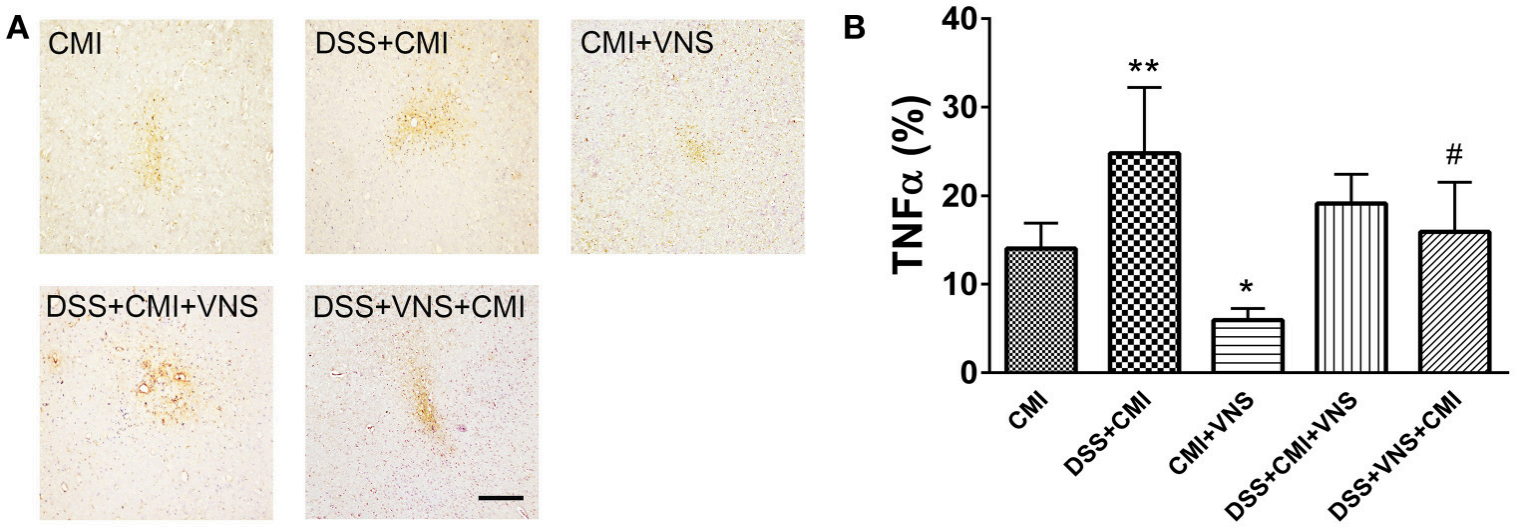
Measurement of TNF-α levels in brain. **(A)** Immunohistochemical staining of TNF-α in the cortex of the CMI, DSS+CMI, CMI+VNS, DSS+CMI+VNS, and DSS+VNS+CMI mouse groups (100×). **(B)** Histograms comparing the area percentage of TNF-α in the cortex (*n* = 8 per group). ^*^P ≤ 0.05; ^**^P ≤ 0.01 vs. CMI group. #*P* < 0.05 vs. DSS+CMI group. Scale bars, 200 μm.

TNF-α levels increased significantly in the DSS+CMI group compared to those in the CMI group (*P* < 0.01), and the levels were significantly reduced in the CMI+VNS group (*P* < 0.05). Decreased TNF-α expression was observed when VNS were performed during the acute phase of colitis in mice with colitis and CMI. However, our data demonstrated no significant difference between TNF-α levels between the DSS+CMI+VNS and DSS+CMI groups (*P* > 0.05) (Figures 6A,B).

## Discussion

The current study found that VNS reduced CMI volume in mice, in the process of which involved decreased BBB permeation, microglia and astrocytes activation, oxidative stress and proinflammatory cytokine expression. DSS-induced colitis significantly exacerbated microstroke in mice, and VNS alleviated colitis-induced CMI aggravation. Notably, the DSS+VNS+CMI group exhibited smaller infarct volume, less BBB permeation and lower neuroinflammation and oxidative stress than the DSS+CMI+VNS group did, which indicates that in mice with colitis and CMI, VNS treatment during the acute stage of colitis was more effective than that following the CMI onset.

A previous study demonstrated that VNS reduced the infarct volume by 56.3% in transient middle cerebral artery occlusion (MCAO) and by 38.4% in permanent MCAO rat model of focal cerebral ischemia (9). Our results demonstrated that VNS achieved a 53% reduction in the infarct volume in a mouse model of CMI, which indicates that VNS-induced neuroprotection extends to CMI.

The BBB is a key component of the neurovascular unit, and its integrity is critical for maintaining brain homeostasis and function. Breakdown of the BBB is an important contributor to CMI pathology and outcome (29), which was supported in our study. VNS exhibited a protective effect on the BBB integrity in our CMI model. Alpha-7 nicotinic acetylcholine receptors (α7 nAChRs) are an essential regulator of the anti-inflammatory effect of VNS (30). Previous studies in traumatic brain injury models revealed that VNS conferred neuroprotection on the BBB integrity (14) and that stimulation of α7nAChRs on splenic macrophages decreased the synthesis/release of proinflammatory molecules, which reduced the BBB permeability (31). Another study used an intracerebral hemorrhage model and suggested that the stimulation of α7 nAChRs on brain endothelial cells exerted protective effects on the BBB integrity via phosphatidylinositol 3-kinase-Akt–induced inhibition of glycogen synthase kinase-3β and β-catenin stabilization (32). Proinflammatory cytokines, such as TNF-α, are released from endothelial cells, leukocytes and resident cells in the brain following cerebral ischemia injury, and inflammatory cytokines are implicated as mediators of the BBB permeability (31, 33). However, a previous study reported that VNS regulated several I/R-related pathways and suppressed inflammatory cytokine synthesis (13). Our study also observed a marked downregulation of TNF-α levels in the brains of CMI mice. Therefore, VNS may preserve the BBB integrity in our mouse CMI model via the modulation of α7nAChRs and inhibit the expression of inflammatory cytokines. However, the exact mechanism requires further investigation.

Inflammation is a key contributor to ischemic cerebral injury, including CMI and Alzheimer's disease. CMI contributes to the cognitive decline in individuals at high risk for Alzheimer's disease (34). Our study found that reactive microglia infiltrated the ischemic core and that reactive astrocytes existed in the peri-infarct area in mice with CMI. However, many studies reported that VNS could alleviate ischemic cerebral lesions and Alzheimer's disease via anti-inflammatory effects (10, 13, 35) involving several possible mechanisms. First, the VNS could activate α7nAChRs expressed on microglia and astrocytes in a rat model of acute cerebral I/R injury for ischemia insult to cause a significant reduction in α7nAChRs on the surfaces of microglia and astrocytes, thus suppressing the expression of inflammatory cytokines (13, 15, 36). The stimulation of α7nAChRs also conferred neuroprotection via inhibition of the activation and infiltration of inflammatory cells (37). Second, VNS could reverse the morphological signs of microglial aging and activation in a murine model of Alzheimer's disease (35). Third, VNS could regulate the neuroinflammatory response following cerebral I/R injury via upregulation of the expression of peroxisome proliferator-activated receptor γ and subsequently suppress the synthesis and secretion of proinflammatory cytokines (38). Our results consistently suggest that VNS influenced the activation of microglia and astrocyte and the expression of proinflammatory cytokines. Therefore, anti-inflammatory effects may be involved in the neuroprotection of VNS in CMI model.

Oxidative stress, such as the deposition of NT, is also a primary event leading to brain damage after cerebral ischemia, including CMI, and inhibition of oxidative stress is neuroprotective (29, 39). Oxidative stress was high in the ischemic core and surrounding penumbra area in the present study, which is consistent with a previous report (39). Recent studies suggest that VNS plays an important role in the inhibition of oxidative stress. For example, VNS exerts neuroprotective effects against ischemia/reperfusion (I/R) via modulation of miR-210 activity, which is an important microRNA that is regulated by hypoxia-inducible factor and Akt-dependent pathways (15). Furthermore, α7nAChR agonist treatment attenuated oxidative stress in mouse models of MCAO and tibia fracture via modulation of anti-oxidant gene expression (36). However, our results demonstrated that VNS significantly modulated the oxidative stress response, which was associated with the downregulation of NT deposition.

Previous reports found that intestinal dysfunction aggravated poststroke neuroinflammation and infarct volumes (3, 4). We also found that DSS-induced colitis significantly exacerbated the infarct volume of CMI in mice, which may be due to acceleration of BBB permeation and aggravation of the inflammatory response and oxidative stress. On the other hand, previous studies suggested that the levels of circulating Th17 and Th1 cells were significantly increased in patients with ulcerative colitis (40), which aggravated the inflammatory damage and outcome in stroke model (4). That would be a alternative mechanism how colitis aggravated the ischemic stroke, However, whether similar mechanism exists in CMI yet remains to be studied. In addition, our data showed that DSS-induced colitis aggravated CMI without the influence of blood flow. Proinflammatory cytokines are key mediators in the pathological process of IBD (16). Proinflammatory factors, such as TNF-α, enter the brain through the disrupted BBB after the onset of CMI and subsequently cause the activation of more microglia to produce more inflammatory factors, which may further trigger secondary reactions, thus beginning a self-propelling and vicious cycle of neuronal damage (41). Previous studies demonstrated that VNS protected against intestinal inflammation via inhibition of the synthesis/release of inflammatory cytokines (16–19). The amount of TNF-α was reduced significantly in the DSS+VNS+CMI group compared to that of the DSS+CMI group, which protected against the colitis-induced CMI aggravation and included BBB disruption, neuroinflammatory glial overreaction and oxidative stress. However, TNF-α levels were not reduced significantly in the DSS+CMI+VNS group compared with those of the DSS+CMI group because VNS did not act effectively against the colitis at this time point, and the excess of inflammatory mediators produced from colitis entered the brain via the disrupted BBB after CMI onset to cause a serious of secondary ischemic brain injury. Therefore, VNS exhibited a weaker protective role in CMI than that in the DSS+VNS+CMI group. Our data indicate that VNS reduced the exacerbation of colitis-induced CMI, and therapy for colitis was critical in this condition.

There are several limitations in our study. We did not monitor the effect of colitis and VNS on the circulating factors and cells, which will be studied in our future study. Also, it was reported that estrogen provides neuroprotection against cerebral ischemic injury by activating estrogen receptors (42) and female mice developed less severe colitis than male mice in DSS-induced colitis, and this protection seems to be mediated by estradiol (43), which might be a potential effect in our study. It was a limitation that the stage of estrous cycle in individual mice was not determined here. Besides, we only used one VNS parameter following previous studies, we will explore the protective effect of different parameters on the ischemic stroke.

In conclusion, our results indicated that VNS alleviated CMI and this neuroprotection was associated with the suppression of BBB permeability, neuroinflammation, oxidative stress. Also, we concluded that VNS alleviated colitis-induced microstroke aggravation. Therefore, VNS may provide a therapeutic opportunity to improve the outcome of microstroke patients, and VNS performed in the acute phase of IBD may improve the therapeutic treatment of patients with IBD and CMI.

## Ethics statement

This study was carried out in accordance with the guidelines of Sun Yat-sen University (Guangzhou, China) Committee on the Care and Use of animals.

## Author contributions

XC, XH, ZL, ZP, and RH: designed the experiment; XC, XH, YF, FL, and TS: performed the experiment; XC, XH, SL, ZL, and ZP: analyzed the data; XC, XH, ZL and ZP: wrote the manuscript.

### Conflict of interest statement

The authors declare that the research was conducted in the absence of any commercial or financial relationships that could be construed as a potential conflict of interest.
